# Supplementary material for: Cost efficiency of commercial banks in Ethiopia: Does financial technology matter?
Source: PLoS One. 2025 Jan 13;20(1):e0317226. doi: 10.1371/journal.pone.0317226 (PMC11729950; doi:10.1371/journal.pone.0317226)
Supplement: Attachment — Submitted filename: Review_2.pdf [file pone.0317226.s002.pdf]

As we have little information on the Ethiopian banking sector in general, any paper with the aim of shedding light on issues of efficiency, operational costs, and profitability triggers attention in the international literature. Also, conducting research on the impact of financial technologies in emerging markets is of crucial importance. The aims, objectives, and the methodologies used in the paper are clear and easy to follow. The recommendations are indeed to be followed by the commercial banks.

The weakest points of the paper are easy to be detected. Firstly, there is no solid literature review on the adoption of financial technology by commercial banks. A number of authors measure the impact, the effects on costs, profitability, earnings, market capitalisation, etc. It would be highly important to show the findings of these papers in order to better understand the Ethiopian room for manoeuvre in the overall transformation. It would be the best to compare the impacts in case of the advanced banking sector and in case of the emerging country banks. Secondly, the reviewer has to highlight that there is no overview of the methodologies used when measuring the impact. We have a clear view on the methods used in this paper but there is little information on what others use. With the help of a quick overview, we could get a better picture on why the authors use the method mentioned and how it differs from the methods used by others.

All in all, the reviewer has a high opinion of the scientific contribution of the paper but corrections are need as it has been mentioned above.
